# Slowed alpha oscillations and percept formation in psychotic psychopathology

**DOI:** 10.3389/fpsyg.2023.1144107

**Published:** 2023-06-14

**Authors:** Scott R. Sponheim, Joshua J. Stim, Stephen A. Engel, Victor J. Pokorny

**Affiliations:** ^1^Minneapolis VA Health Care System, Minneapolis, MN, United States; ^2^Department of Psychiatry and Behavioral Sciences, University of Minnesota, Minneapolis, MN, United States; ^3^Department of Psychology, University of Minnesota, Minneapolis, MN, United States

**Keywords:** alpha rhythm, oscillation, psychosis, schizophrenia, binocular rivalry, cognition

## Abstract

**Introduction:**

Psychosis is in part defined by disturbances in perception. Recent investigations have implicated the speed of alpha oscillations observed in brain electrical activity as reflective of a sampling rate of the visual environment and perception. Although both slowed alpha oscillations and aberrant percept formation are evident in disorders of psychotic psychopathology such as schizophrenia it is unclear whether slow alpha accounts for abnormal visual perception in these disorders.

**Methods:**

To examine the role of the speed of alpha oscillations in perception in psychotic psychopathology we gathered resting-state magneto-encephalography data from probands with psychotic psychopathology (i.e., schizophrenia, schizoaffective disorder, and bipolar disorder with a history of psychosis), their biological siblings, and healthy controls. We appraised visual perceptual function without the confound of cognitive ability and effort through the use of a simple binocular rivalry task.

**Results:**

We found a slowed pace of alpha oscillations in psychotic psychopathology that was associated with longer percept durations during binocular rivalry, consistent with the assertion that occipital alpha oscillations govern the rate of accumulation of visual information used to generate percepts. Alpha speed varied widely across individuals with psychotic psychopathology and was highly stable across several months indicating that it is likely a trait characteristic of neural function that is relevant to visual perception. Finally, a lower speed of alpha oscillation was associated with a lower IQ and greater disorder symptomatology implying that the effects of the endogenous neural oscillation on visual perception may have wider consequences for everyday functioning.

**Discussion:**

Slowed alpha oscillations in individuals with psychotic psychopathology appear to reflect altered neural functions related to percept formation.

## Introduction

Given that the external world provides essentially the same visual information to all humans, differences in how stimuli are perceived are due to internal sensory processes that vary across people. In psychotic psychopathology these internal sensory processes produce hallucinations and illusions (Silverstein and Lai, 2021). Although a variety of visual perceptual abnormalities have been documented in psychotic psychopathology (Silverstein and Keane, 2011), it remains unclear what endogenous sensory processes give rise to disturbed perception. Mathematical and associated neurobiological formulations of the basis of disturbed perception in psychotic psychopathology have yet to implicate a specific neural process that accounts for visual perceptual disturbance in disorders such as schizophrenia (Kafadar et al., 2020, 2022). Recently, Torralba Cuello et al. (2022) provided evidence that occipital alpha oscillations measured by electroencephalography (EEG) reflect the accumulation of visual information and facilitate the development of perceptual representations. Their findings supported the notion that alpha oscillations provide a window for re-appraisal of visual input to produce one of several competing perceptual representations of objects. Competition between visual representations, or percepts, is most apparent with ambiguous stimuli that yield more than one representation (e.g., Necker cube, face-vase illusion). If alpha oscillations define temporal windows for visual perception, a faster pace of oscillations is likely to support more efficient processing of sensory information and the formation of percepts.

Researchers have shown that people with psychotic psychopathology such as schizophrenia have difficulty in adaptively forming percepts from external stimuli (Feigenson et al., 2014; Spencer and Ghorashi, 2014; Keane et al., 2016; McCleery et al., 2020; Longenecker et al., 2021; Osborne et al., 2022). Also, the pace of the alpha oscillations is slowed in people with schizophrenia, which is typically measured as the frequency of the alpha peak in the frequency spectrum of the resting EEG (Yeum and Kang, 2018; Murphy and Öngür, 2019; Castelluccio et al., 2020). We have shown that lower alpha peak frequencies are associated with lower intelligence scores that may be mediated by the ability to distinguish visually degraded images of numerals from one another, thereby suggesting that the pace of alpha oscillations is relevant to perceptual anomalies and tied to cognitive deficits in schizophrenia (Ramsay et al., 2021). Yet, the association between abnormalities in an endogenous neural oscillation such as alpha and endogenous perceptual phenomena remains unexamined in psychotic psychopathology. If a reduced pace of alpha oscillations observed in psychotic psychopathology is related to endogenous aspects of perception (i.e., internal sensory processes) it would suggest that slowed accumulation of visual information likely compromises the formation of perceptual representations in the disorder.

In the present study we appraised visual perceptual function without the confound of cognitive ability and effort through the use of a simple binocular rivalry task. We also acquired magneto-encephalography (MEG) during eyes closed and eyes open resting states to measure the individual alpha peak frequency (IAPF) to assess the pace of alpha oscillations for each participant. Fluctuating perceptions when viewing stable but ambiguous objects such as those used in binocular rivalry are a reflection of endogenous processes important to perception. If the pace of alpha oscillations reflects the period of time for reappraisal of perceptual representations, individuals with psychotic psychopathology would exhibit an association between alpha peak frequency and the duration of percepts during the binocular rivalry task.

## Materials and method

### Participants

The study sample included 34 probands with a history of psychosis (PRO) (i.e., delusions, hallucinations, or formal thought disorder) and were diagnosed with schizophrenia, schizoaffective disorder, or bipolar disorder, 10 first-degree biological siblings of PRO (REL), and 28 healthy control participants (CONs). All PRO and CONs were recruited from the Minneapolis VA Medical Center, University of Minnesota, and from community postings. Exclusion criteria were 1) English not being a primary language, 2) a charted or reported IQ of less than 70, 3) drug or alcohol dependence within the previous 6months, or current drug or alcohol abuse, 4) current or past central nervous system disease, 5) head injury with skull fracture or loss of consciousness of 30 minutes or more, 6) presence of a condition that would render study measures difficult or impossible to administer or interpret (e.g., corrected visual acuity worse than 20/30, significant astigmatism, cataract, or visual field loss), 7) age less than 18 years or greater than 50 years, 8) significant tardive dyskinesia, and 9) a history of electro-convulsive therapy. For each eligible PRO who passed exclusion criteria a list of all live-born first-degree biological siblings was compiled. RELs were contacted by mail and a follow-up telephone call to determine their interest and eligibility for participating in the study. Potential CONs were excluded if they had a personal history or a family history (i.e., first degree biological relative) of psychotic symptomatology or major affective episodes requiring hospitalization.

In order to assess symptom domains and inform diagnoses in PROs, RELs, and CONs, a trained and supervised research staff or clinical psychology graduated student completed the Structured Clinical Interview for DSM-IV (SCID-I) (First, 2015) with the participant. To ensure complete coverage of psychotic symptomatology the Psychosis Module of the Diagnostic Interview for Genetic Studies (DIGS) (Nurnberger, 1994) was administered in place of the SCID Psychosis section. A trained research assistant also conducted a medical chart review when records were available to obtain collateral information about the subject’s functioning, and past and present symptomatology. From the interview and chart information study staff completed ratings of symptomatology using the Scale for the Assessment of Negative Symptoms (SANS) (Andreasen, 2014) and the Scale for the Assessment of Positive Symptoms (SAPS) (Andreasen, 2018). Interviewers also made ratings using the Brief Psychiatric Rating Scale 24-item version (BPRS) (Ventura et al., 2000) to quantify mood and other behavioral characteristics of clinical state. Totals for global item ratings on the SANS and SAPS were used to characterize psychotic symptomatology while total and factor scores on the BPRS (Wilson and Sponheim, 2014), were used to summarize a mood and psychotic symptoms of participants. Finally, the Schizotypal Personality Questionnaire (SPQ) (Raine, 1991) and Sensory Gating Inventory (Hetrick et al., 2012) were completed by participants to quantify subtle psychotic characteristics and perceptual distortions experienced in their everyday lives. IQ was measured using the Matrix Reasoning and Similarities subtests of the Wechsler Adult Intelligence Scale IV (Wechsler, 2012; Denney et al., 2015).

Table 1 includes the participant characteristics. PRO were lower than other groups in estimated IQ and years of education, and higher in all symptom domains except mania consistent with the sample of PRO being largely composed of stable outpatients. Chlorpromazine (CPZ) equivalents were computed for the 26 out of 34 PRO (76%) who were currently taking antipsychotic medications at the onset of the study. All participants had the capacity to understand the study procedures and completed an informed consent process in accordance with the Declaration of Helsinki. Compensation was provided commensurate to the visit time and procedures. The University of Minnesota and Minneapolis VAHCS Institutional Review Boards both provided approval and monitoring of the study.

**Table 1:** Participant characteristics.

Variables	Psychosis (*n* = 34)	Relatives (*n* = 10)	Controls (*n* = 28)	Test Statistic	*value of p*	Post-hoc Contrasts
Age	36.50 ([32.60–40.50])	35.40 ([28.30–42.50])	35.75 ([32.80–38.70])	*F* (2,69) = 0.074	0.928	**–**
Female, *n (%)*	14 (41%)	6 (60%)	13 (46%)	*χ^2^*(2) = 1.11	0.574	**–**
IQ	97.50 ([93.50–101.5])	106.0 ([98.73–114.9])	111.0 ([107.0–115.0])	*F* (2,68) = 11.88	<0.001	Pro < Con
Years of Education	14.60 ([13.80–15.40])	15.70 ([14.30–17.10])	17.20 ([16.40–17.90])	*F* (2,69) = 11.35	<0.001	Pro < Con
*Total Symptoms (BPRS)*	38.00 ([34.25–50.25])	29.50 ([26.75–31.75])	28.00 ([26.00–29.00])	*χ2*(2) = 42.37	<0.001	Pro > Con, Pro > Rel
Positive Psychosis	7.50 ([6.25–11.75])	5.00 ([5.00–5.00])	5.00 ([5.00–5.00])	*χ2*(2) = 37.73	<0.001	Pro > Con, Pro > Rel
Negative Psychosis	4.00 ([3.00–5.00])	3.00 ([3.00–3.00])	3.00 ([3.00–3.25])	*χ2*(2) = 8.30	0.015	Pro > Con, Pro > Rel
Disorganization	7.00 ([5.25–8.00])	5.00 ([4.25–5.75])	5.00 ([4.00–5.25])	*χ2*(2) = 21.73	<0.001	Pro > Con, Pro > Rel
Depression-anxiety	6.50 ([5.00–10.75])	3.50 ([3.00–6.75])	4.00 ([3.00–5.00])	*χ2*(2) = 14.44	<0.001	Pro > Con, Pro > Rel
Mania-excitement	3.00 ([3.00–3.00])	3.00 ([3.00–3.00])	3.00 ([3.00–3.00])	*χ2*(2) = 5.67	0.059	**–**
*Total Schizotypal Traits (SPQ)*	35.50 ([18.00–43.00])	9.00 ([2.50–15.75])	9.00 ([4.50–14.25])	*χ2*(2) = 31.47	<0.001	Pro > Con, Pro > Rel
Cognitive Perceptual	13.50 ([7.25–18.00])	1.50 ([1.00–4.25])	1.00 ([0.00–2.25])	*χ2*(2) = 34.86	<0.001	Pro > Con, Pro > Rel
Interpersonal	15.00 ([8.00–21.00])	2.50 ([2.00–7.75])	5.00 ([1.75–8.50])	*χ2*(2) = 21.03	<0.001	Pro > Con, Pro > Rel
Disorganized	7.50 ([4.25–11.00])	3.00 ([0.00–5.00])	2.00 ([1.00–4.00])	*χ2*(2) = 21.11	<0.001	Pro > Con, Pro > Rel
*Sensory Gating Inventory (SGI)*	88.00 ([63.2–106.2])	39.00 ([33.2–52.5])	42.00 ([29.0–68.2])	*χ2*(2) = 23.13	<0.001	Pro < Con
*Total Negative Symptoms (SANS)*	5.50 ([4.00–8.75])	–	**–**	**–**	**–**	**–**
*Total Positive Symptoms (SAPS)*	3.00 ([1.25–6.75])	–	**–**	–	–	–
*CPZ Equivalents**^a^*	241.5 ([29.50–549.1])	–	**–**	**–**	**–**	**–**

Psychosis probands were individuals with schizophrenia (*N* = 12), schizoaffective disorder (*N* = 11), and bipolar disorder (*N* = 11). Relatives were siblings of probands. Mean and confidence intervals are provided for demographic characteristics, while median and interquartile range are provided for the symptom, cognition, and medication measures. IQ was estimated from two subtests of the WAIS-IV. The Brief Psychiatric Rating Scale (BPRS) reflected current clinical symptomatology. The Schizotypal Personality Questionnaire (SPQ) measured psychotic-like experiences, the Scale for the Assessment of Negative Symptoms (SANS) and Positive Symptoms (SAPS) measured severity of the two broad domains of psychotic symptomatology. We tested for group differences in mean age, estimated IQ, and years of education using one-way ANOVAs and obtained post-hoc contrasts for these variables using one-tailed t-tests. We used a Pearson’s Chi-squared test to test for group differences in gender proportion. Lastly, due to skewness in our clinical data, we applied Kruskal-Wallis to test for group differences in BPRS, SPQ, and SGI, and used Wilcoxon Rank Sum tests to obtain post-hoc contrasts for each of these measures.

a 26 out of 34 PRO (76%) reported current usage of antipsychotics at study onset. CPZ = Chlorpromazine.

### Resting state MEG acquisition and processing

MEG data were collected at 1017.25 Hz using a 248-sensor axial gradiometer MEG system (Magnes 3600WH, 4D-Neuroimaging), housed within an electromagnetically-shielded room at the Minneapolis VA Medical Center. Environmental noise was removed from the data by relating signals from separate reference channels to cortical channels. Sensor configuration and head position were obtained using a spatial digitizer. Participants completed six 45 s blocks of resting state alternating between eyes open and closed. We gathered both eyes open and eyes closed resting states in psychotic patients to appraise the stability of IAPF across the two conditions that are commonly used in resting state studies of psychopathology. For the eyes open condition participants blinked naturally and were instructed to fixate on a black dot in the middle of a computer monitor. PRO were invited to return for MEG and binocular rivalry assessments 3 months after the date of their initial data collection. Eighteen PRO completed these follow-up assessments.

All MEG data processing and analyses were conducted in Matlab (The MathWorks, Inc. Natick, MA, United States). Resting data were high-pass filtered at 0.1 Hz, low-pass filtered at 110hz and resampled to 250 Hz. EEGLAB’s pop_clean_rawdata function was used to detect and remove bad time segments and electrodes (FlatLineCriterion = 5, ChannelCriterion = 0.7, WindowCriterion = 0.25, WindowCriterionTolerance = −Inf – 7). Independent component analysis (ICA) was then conducted using EEGLAB’s pop_runica function with the ‘extended’ option set to 1. ICs were labeled automatically using pop_icflag and ICs were rejected when labeled as muscle, eye, heart, line noise or channel noise with greater than 80% confidence. An average of 4.16 components were removed per subject (SD = 4.34). Finally, bad sensors deleted prior to ICA were interpolated via spherical splines. An average of 16.19 sensors (out of 248 total sensors; i.e. around 6% of the sensors) were removed per subject (SD = 16.61).

### IAPF measurement

Following individual subject pre-processing, eyes closed and eyes open resting data were separately submitted to an automatic IAPF peak detection algorithm (Corcoran et al., 2018). Only sensors from the back of the head were submitted for peak detection (see Supplementary Figure S1). The parameters for the peak detection algorithm were as follows: cmin = 3, frange = 1–20, w = 7–13, Fw = 11, k = 5. To distinguish between competing peaks within the alpha band frequency range (i.e., 7-13hz), the mean difference (mdiff) parameter was set to the default value of 20% such that a dominant peak needed to be at least 20% larger than competing peaks. These parameters were chosen in accordance with the recommendations of Corcoran et al. (2018).

### Binocular rivalry paradigm

**Figure 1:**
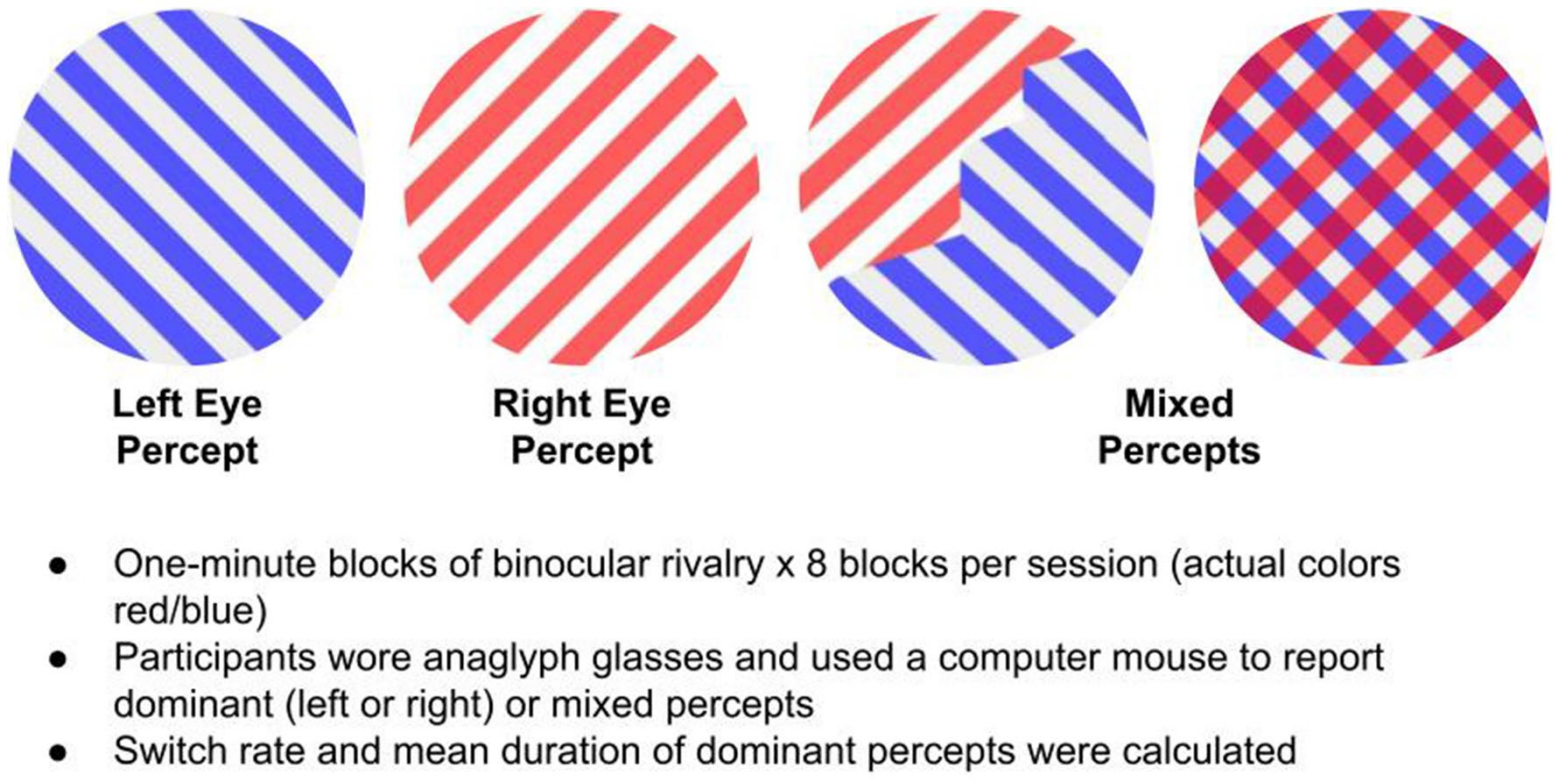
Binocular rivalry task.

Each participant completed eight 1-min runs of the binocular rivalry task, with a 30 s break between each block. Participants pressed one of three buttons to report if they perceived +45 (right eye), −45 (left eye), or mixed gratings (Figure 1 depicts the binocular rivalry stimuli that were viewed through anaglyph glasses). Participants were instructed to use computer mouse buttons to report switches in perceptual states (either dominant or mixed). They received the following instructions, “This task involves looking at a stimulus that consists of a set of blue gratings overlaid on a set of red gratings. When you view this stimulus through red-blue anaglyph glasses, you will experience a perceptual illusion, such that your perception will continually switch between gratings, so that at one moment you might only see the left-leaning gratings, but at the next moment you might only see the right-leaning gratings, or perhaps a mixture of the two. Your job for this task is to tell me what you are seeing, as you are seeing it. You will report what you are seeing by using a computer mouse. Press the left mouse button when you see *only* the left-leaning gratings, press the right mouse button when you see *only* the right-leaning gratings, and press the scroll button when you see a mixture of the two. In most cases, the mixture will not appear as one grating perfectly overlaid on top of the other. Rather, the mixture will likely appear as a ‘piecemeal’ combination of the two gratings.”

### Percept duration measurement

For each participant, we computed percept durations (PD) by measuring the amount of time between each reported perceptual switch. Due to variability in how participants might classify mixed percepts, we decided to exclude mixed PDs from these analyses and only considered dominant PDs (i.e., +45 and − 45). Previous research suggests that perceptual durations in binocular rivalry are lognormally distributed within individuals (Ye et al., 2019). To assess the probability that the PDs in our sample were derived from a lognormal distribution, we conducted Kolmogorov–Smirnov goodness-of-fit tests, which yielded a mean value of p of 0.4260. We then fit a lognormal distribution to each participant’s PD sample using the *lognfit* function in MATLAB (*source*), which used the participant’s PD sample to generate maximum likelihood estimates for the two parameters (location: μ , scale: σ ) of the lognormal probability density function (Eq. 1). We used these estimated parameters to compute the mean of the corresponding fitted distribution for each participant (Eq. 2).


(1) $$f\left(x\right)=\frac{1}{\sqrt{2\pi \sigma x}}\exp\left(\frac{-{\left(\ln\;x-\mu \right)}^{2}}{2\sigma^{2}}\right)$$

(2) $$\mathrm{Mean}\left(x\right)=\exp\left(\mu -\frac{\sigma^{2}}{2}\right)$$

### Statistical analyses

All statistical analyses were completed using Rstudio Version 2022.12.0. One-tailed Welch’s two-sample t-tests were used to test for differences between PRO and CON in eyes open IAPF, eyes closed IAPF and perceptual duration. Relatives were not compared to other groups given their small sample size. Two Pearson-product moment correlations were computed to quantify the association between the two IAPF conditions and PD across groups. We also computed these correlations within groups (again we chose not to quantify the association within the REL group due to low sample size). The test–retest reliability (or stability) of IAPF and PD was quantified using both the Pearson product moment correlation and the Intraclass correlations (ICC[2,1]) which is a two-way random effects, absolute agreement, single rater/measurement model. We interpreted the quality of the ICC using the guidelines as described in Cicchetti (1994) such that ICC < 0.4 was poor, ICC between 0.40 and 0.59 was fair, between 0.60 and 0.74 was good, and between 0.75 and 1 was excellent. We also assessed the Pearson correlation, across groups, between 1) IAPF and BPRS total ratings and 2) IAPF and IQ. Similar correlations were computed within the PRO group only. Finally, we ran two multiple regression models in which group membership and eyes-closed IAPF were the predictors and the dependent variables were IQ and BPRS total scores.

## Results

### Group contrasts

**Figure 2:**
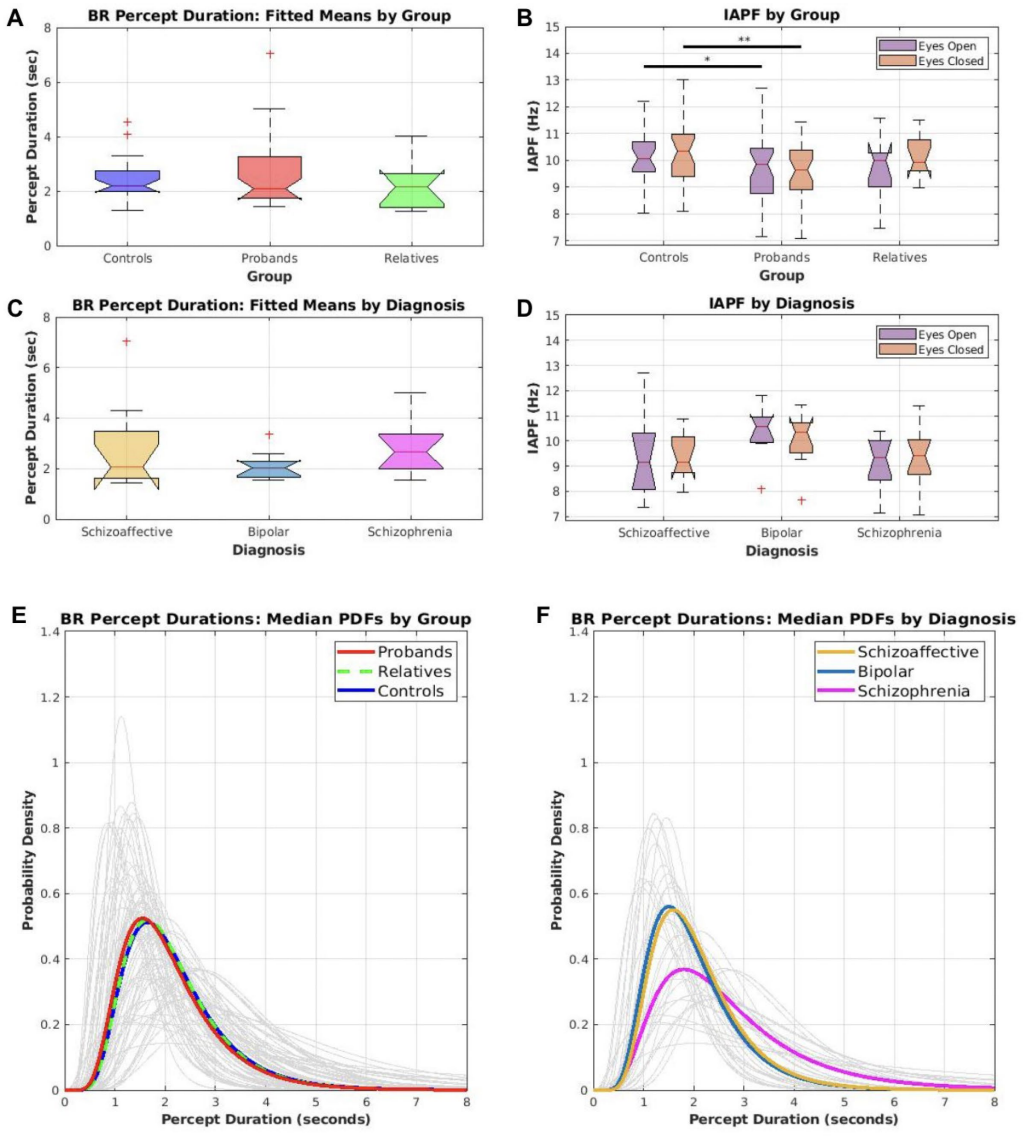
Distributions for IAPF and percept duration by group and diagnosis **(A–D)** Boxplots show the spread of mean percept duration (PD) during binocular rivalry (BR) and resting state IAPF by group and diagnosis. Comparison tests were only conducted between controls and probands; sample sizes for relatives and individual diagnostic groups were too small to test for significant differences, but the boxplots for all groups were displayed for the sake of completeness. **(A)** The distributions of mean percept durations for the control, relative, and proband groups. **(B)** The distributions of IAPF for both resting conditions (eyes open and eyes closed) within the control, relative, and proband groups. Probands had significantly lower IAPF for eyes open (*p < 0.05) and eyes closed (**p < 0.01) compared to controls. **(C)** The distributions of mean percept durations for probands only, broken down by diagnosis. **(D)** The distributions of IAPF for both resting conditions for probands only, broken down by diagnosis. **(E–F)** A lognormal probability density function was fitted to each participant’s set of reported percept durations and plotted above (light gray lines). **(E)** Summary distributions were computed for controls, relatives, and probands using the median parameters within each group (bold, colored lines). **(F)** Summary distributions were computed for each diagnostic group using the median parameters within each diagnosis (bold, colored lines).

Boxplots A–D of Figure 2 depicts the distributions of IAPF and PD for PRO, REL, and CONs. Also included are the PRO separated into the diagnostic groups of schizophrenia, schizoaffective disorder, and bipolar disorder. Table 2 includes the mean PD and IAPF for PRO, REL, and CONs. As a group PRO showed lower IAPF than CONs in both eyes closed (*t* (56.7) = 2.62, *p* = 0.006, Cohen’s d = 0.68; PRO M = 9.59, SD = 1.09; CON M = 10.3 SD = 1.01) and eyes opened (*t* (50.82) = 1.98, *p* = 0.026, Cohen’s d = 0.53; PRO M = 9.65, SD = 1.33; CON M = 10.27, SD = 1.03) conditions. Thus, psychotic psychopathology was associated with a slower speed of alpha oscillations. Percept duration failed to significantly differ between PRO and CONs (*t* (56.89) = −1.22, *p* = 0.114, Cohen’s d = −0.29) although numerically the PD was longer in PRO (PRO M = 2.7, SD = 1.28; CON M = 2.38, SD = 0.77). Additionally, supplemental analyses comparing mean PD and PD variance between controls, relatives, and the three proband subgroups (i.e., schizophrenia, bipolar, and schizoaffective) did not reveal any significant differences, although numerically the schizophrenia subgroup displayed longer mean PD and greater PD variance (see Supplementary Table S1). Therefore, the schizophrenia group tended to exhibit deviations toward prolonged percept durations.

**Table 2:** Percept duration (PD) and individual alpha peak frequency (IAPF) means and standard deviations for proband, relative, and control groups.

	CON	PRO	REL
Perceptual duration (Fitted Mean)	2.38 (0.77)	2.7 (1.28)	2.18 (0.83)
IAPF eyes open	10.27 (1.03)	9.65 (1.33)	9.65 (1.13)
IAPF eyes closed	10.3 (1.01)	9.59 (1.09)	10.19 (0.78)

### Association of IAPF with percept duration

**Figure 3:**
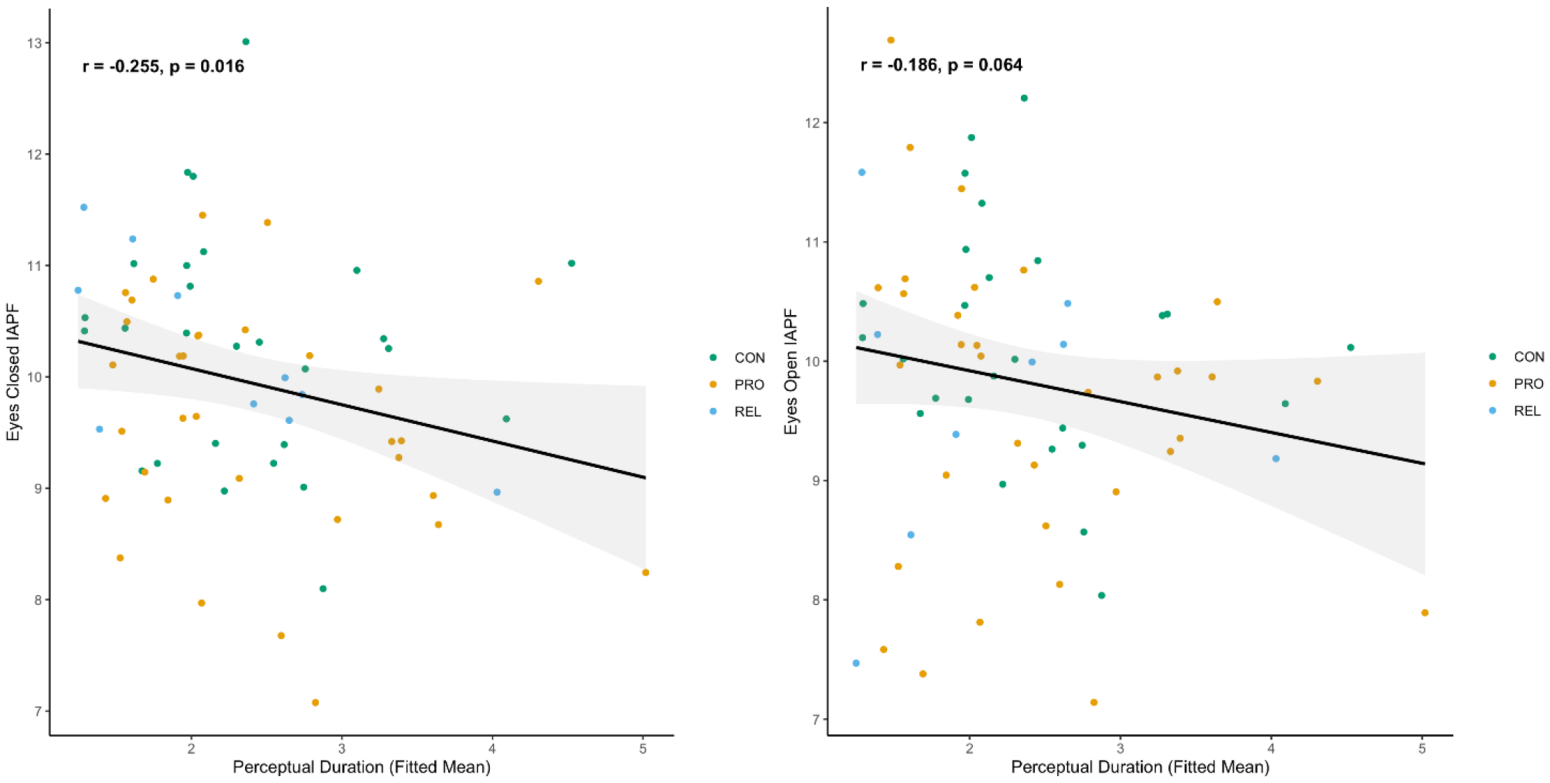
IAPF is predictive of percept duration during binocular rivalry, scatterplots of IAPF derived from resting state MEG against percept duration during binocular rivalry for psychosis probands, biological relatives, and control participants. Each dot represents one individual. Similar plots with separate regression lines for each group available in Supplementary material. Left Panel association of eyes closed IAPF with percept duration, Right Panel association of eyes open IAPF with percept duration.

To determine whether the pace of alpha oscillations was associated with the period of time for which percepts tended to exist before a change to a mixed or competitive percept, we examined the association of IAPF with PD. Slower alpha (i.e., lower IAPF) during eyes closed rest was associated with longer PDs (*r* = −0.25; *p* = 0.016). Eyes open rest IAPF showed a similar trend, but the value of p did not fall below the 0.05 alpha threshold (*r* = −0.18; *p* = 0.064). Scatterplots of these associations are depicted in Figure 3. Within CON there were no significant associations between IAPF and PD (eyes closed *r* = −0.109, *p* = 0.294; eyes open *r* = −0.218, *p* = 0.142). Within PRO, the associations were similarly not significant though the *p* values were less than 0.1 and the effect sizes were generally of the same size and direction as the across group correlations (PRO: eyes closed *r* = −0.25, *p* = 0.08; eyes open *r* = −0.23, *p* = 0.09).

### Long-term stability of IAPF and PD in individuals with psychotic psychopathology

**Figure 4:**
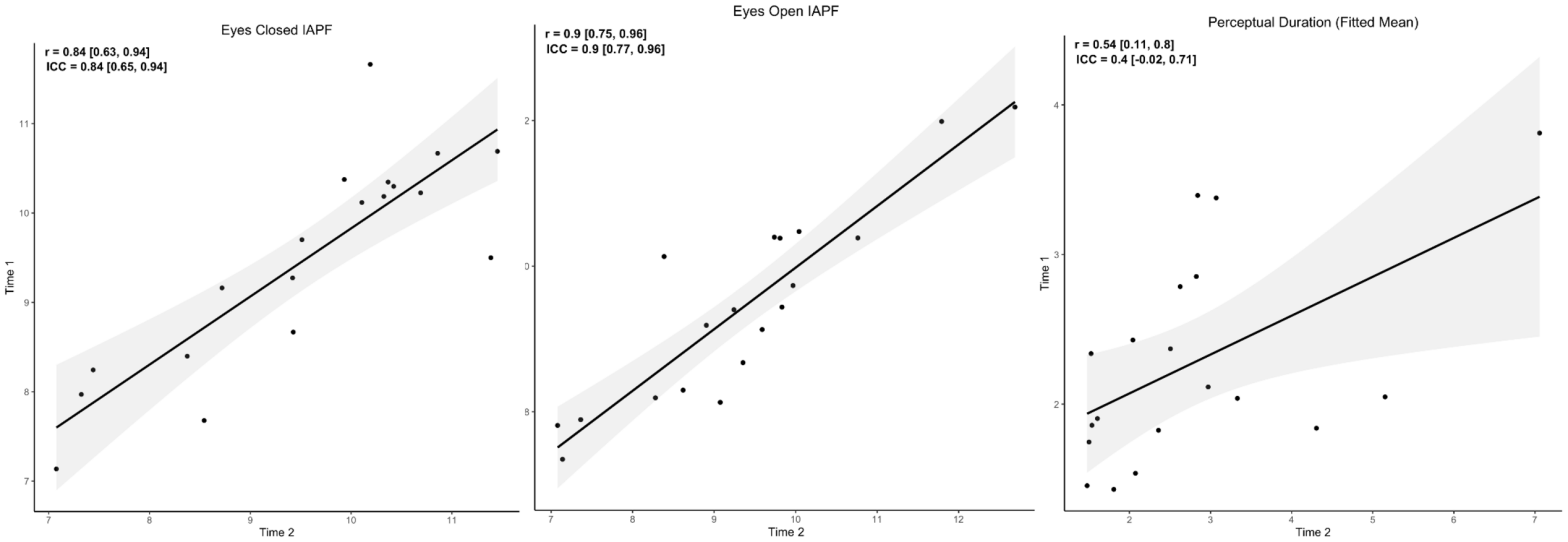
Several-month stability of IAPF and PD. Scatterplots of IAPF and percept duration across two data collection sessions separated by several months. Repeat observations were only performed for psychosis probands. Each dot represents one individual. Left Panel stability of eyes closed resting state IAPF, Middle Panel stability of eyes open resting state IAPF, Right Panel stability of percept duration during binocular rivalry.

Because stable indices are useful for characterizing individual differences that might predict risk for psychotic psychopathology or its severity, we examined the stability of IAPF and PD between baseline and follow-up assessments for PRO. Figure 4 depicts the associations between baseline and follow-up assessments. IAPF showed excellent stability (eyes closed ICC = 0.84, eyes open ICC = 0.9) over the several month period while PD showed fair stability (ICC = 0.4). Thus, both indices have potential to serve as relatively stable trait predictors of aspects of psychotic psychopathology.

### Relationship of IAPF to clinical aspects of psychotic psychopathology

**Figure 5:**
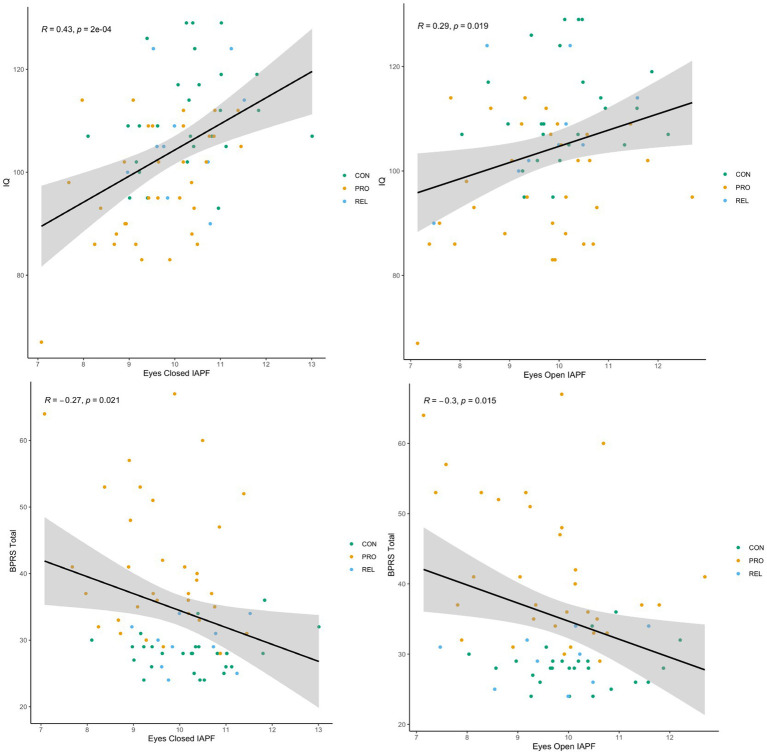
IAPF is predictive of general cognitive functioning and severity of psychopathology. Scatterplots of resting state IAPF against estimated intelligence quotient (IQ) and level of overall psychiatric symptomatology (BPRS Total; BPRS = Brief Psychiatry Rating Scale) for psychosis probands, biological relatives, and control participants. Each dot represents one individual. Similar plots with separate regression lines for each group available in Supplemental material. Left Panels association of eyes closed IAPF with IQ (top) and total symptomatology (bottom). Right Panels association of eyes open IAPF with IQ (top) and total symptomatology (bottom).

Because IAPF was lower in PRO and showed strong stability over a several month period we tested whether the measure of alpha oscillations was related to clinical aspects of the psychotic psychopathology. Figure 5 displays associations of IAPF with IQ and total symptomatology. For both eyes open and eyes closed condition, lower IAPF was associated with lower IQ implying a relationship with overall cognitive functioning (eyes open: *r* = 0.29, *p* = 0.017; eyes closed: *r* = 0.43, *p* < 0.001). Lower IAPF was also predictive of greater symptomatology across all participants as measured by the total score on the BPRS (eyes open: *r* = −0.33, *p* = 0.006; eyes closed: *r* = −0.29, *p* = 0.016). When restricting this analysis to patients only, the eyes closed IAPF association was no longer significant (*r* = −0.21, *p* = 0.23) while the eyes open IAPF association remained significant (*r* = −0.42, *p* = 0.019). To examine the relationship of IAPF with cognition and symptomatology while considering that lower IQ and greater symptoms are largely unique to the PRO sample, we added group membership as an explanatory variable in the regression analysis. When eyes-closed IAPF and group membership were entered simultaneously as predictors in a regression analysis the association between IAPF and IQ persisted (β = 3.57, *t* (66) = 2.88, *p* = 0.01), while the relationship between IAPF and total symptomatology was no longer significant (*β* = −0.66, *t* (67) = −0.73, *p* = 0.47). PD was not significantly associated with IQ (*r* = −0.15, *p* = 0.21) or BPRS total (*r* = −0.035, *p* = 0.77).

## Discussion

In this investigation we examined whether the dominant resting state brain oscillation - the alpha rhythm - was predictive of an endogenous visual function related to percept formation, and whether the association held in psychotic psychopathology. Investigations have repeatedly shown that psychotic psychopathology is characterized by abnormal resting state neural oscillations (Clementz et al., 1994; Venables et al., 2009; Hirano and Uhlhaas, 2021) that include a slower pace of the alpha rhythm (Ramsay et al., 2021). Specifically, we found that the slowed pace of alpha oscillations noted in psychotic psychopathology was associated with longer percept durations measured using a binocular rivalry task. When restricted to individuals with psychotic psychopathology the association was just as strong as the analysis across groups but was a statistical trend due to the smaller sample size. The association between IAPF and PD we identified is a replication of associations reported in normative samples (Katyal et al., 2019; Torralba Cuello et al., 2022). Results of the present study are consistent with the assertion that occipital alpha oscillations govern the rate of accumulation of visual information used to generate percepts (Torralba Cuello et al., 2022). Slowed alpha oscillations in individuals with psychotic psychopathology appear to reflect altered neural functions related to percept formation.

Importantly, we found that the alpha peak frequency varies widely across individuals with psychotic psychopathology, but is also highly stable across several months indicating that the IAPF is likely a trait characteristic of neural function that is relevant to visual perception. Finally, lower IAPF was associated with a lower IQ implying that the possible effects of the endogenous neural oscillation on visual perception have wider consequences for cognition. This association replicates a previous report of IAPF predicting IQ in a larger sample that included evidence that the association was mediated by visual sustained attention (Ramsay et al., 2021). Lower IAPF was also associated with more psychiatric symptomatology consistent with slowed oscillations as an indicator of a general deviation in brain function in psychopathology.

There is an accumulation of evidence for occipital alpha oscillations reflecting the fluctuation of visual perception (Busch and VanRullen, 2010; VanRullen, 2016). Torralba Cuello et al. (2022) hypothesized that alpha plays a key role in the competition between alternative perceptual representations that are present during binocular rivalry. Specifically, they assert that the alpha rhythm provides phasic windows for reevaluating competing percepts, and evidence accumulation for a percept takes place in a phasic manner. They showed that faster switches were present when faster endogenous activity was induced with such effects being restricted to entrainment in the alpha band. This is consistent with the Gating-by-Inhibition hypothesis of Jensen and Mazaheri (2010) which specifies that the alpha rhythm governs the sampling of sensory input *via* peaks of neural excitability and periods of pulsed inhibition. The association of IAPF with PD revealed in the current study and other investigations is consistent with this proposal (Katyal et al., 2019; Torralba Cuello et al., 2022). Researchers have manipulated the IAPF through entrainment *via* stimulus flickers and have provided evidence that the oscillations play a causal role in modulating perception (Grandy et al., 2013; Cecere et al., 2015; Ronconi et al., 2018). Also, the temporal resolution of an individual’s visual perception appears tied to the speed of their alpha rhythm (Samaha and Postle, 2015). Results of the current investigation builds on this work implicating alpha oscillations in visual perception and provides novel evidence that slowed IAPF results in slowed formation of visual perceptual representations. It should be noted that patients were on antipsychotic medications which may have influenced our results; however we did not observe any strong correlations between chlorpromazine equivalent daily dosage and any of our dependent variables of interest (see Supplementary Figure S5).

Individuals diagnosed with schizophrenia and bipolar disorder have been repeatedly shown to exhibit slow percept switching on binocular rivalry tasks (Fox, 1965; Miller et al., 2003; Xiao et al., 2018; Ye et al., 2019). Brain imaging studies have yielded evidence of occipital brain processes being tied to percept formation assessed through binocular rivalry. For instance, using simultaneously recorded EEG and functional magnetic resonance imaging (fMRI) Roy et al. (2017) showed that when percepts were most intact right middle and inferior occipital gyri were more active than during transition between percepts. Transitions between percepts were also associated with decreased activity in primary visual cortex, precuneus and posterior mid-cingulate cortex. Other research has pointed to low-level visual processes underlying the competition of percepts (Petruk et al., 2018). Individuals with heightened genetic liability for schizophrenia or subtle symptom expression consistent with the disorder have also been shown to exhibit compromised percept formation during binocular rivalry (Xiao et al., 2018; Thakkar et al., 2019). Thus, from what is currently known it appears that percept durations in psychotic psychopathology may derive from low-level neural processes reflected in a slow pace of alpha oscillations.

In summary, the present investigation revealed evidence that the slower cycling of the alpha rhythm in psychotic psychopathology is predictive of visual perceptual processes. The association between the speed of alpha oscillations and the duration of percepts during binocular rivalry was across individuals with bipolar disorder, schizoaffective disorder, siblings of individuals with psychotic psychopathology, and healthy controls suggesting that the effect of slowed alpha on visual perception may extend beyond a diagnosis of schizophrenia. A lower IAPF was additionally related to worse cognitive functioning and greater symptomatology consistent with a decreased pace of alpha oscillations serving as an indicator of generalized brain pathology. The stability of IAPF across several months in people with psychotic psychopathology bodes well for the measure to function as a predictor of visual perceptual difficulties and perhaps an indicator of brain abnormalities tied to the development of psychotic psychopathology.

## Data availability statement

The raw data supporting the conclusions of this article will be made available by the authors, without undue reservation.

## Ethics statement

The studies involving human participants were reviewed and approved by Minneapolis VA IRB, University of Minnesota IRB. The patients/participants provided their written informed consent to participate in this study.

## Author contributions

SS, SE, and JS contributed to conception and design of the study. JS and VP acquired, processed, and analyzed data. SS wrote the first draft of the manuscript. SS, JS, and VP wrote sections of the manuscript. All authors contributed to the article and approved the submitted version.

## Funding

This material is the result of work supported with resources and use of facilities at the VA Minneapolis Healthcare System. The research was supported by grants from the National Institutes of Mental Health (R01MH112583) and from the Veterans Health Administration Clinical Science Research and Development Program (01CX001843, 01CX000227) to SS.

## Conflict of interest

The authors declare that the research was conducted in the absence of any commercial or financial relationships that could be construed as a potential conflict of interest.

## Publisher’s note

All claims expressed in this article are solely those of the authors and do not necessarily represent those of their affiliated organizations, or those of the publisher, the editors and the reviewers. Any product that may be evaluated in this article, or claim that may be made by its manufacturer, is not guaranteed or endorsed by the publisher.
