# Supplementary material for: Slowed alpha oscillations and percept formation in psychotic psychopathology
Source: Front Psychol. 2023 Jun 14;14:1144107. doi: 10.3389/fpsyg.2023.1144107 (PMC10322206; doi:10.3389/fpsyg.2023.1144107)
Supplement: Supplementary file 1 [file Data_Sheet_1.docx]

Supplementary Materials

| ***Supplemental Table 1a: Sample distribution of mean percept durations*** | | | | | | | | |  |
| --- | --- | --- | --- | --- | --- | --- | --- | --- | --- |
| **Group** | **n** | **minimum** | **lower quantile** | **median** | **upper quantile** | **maximum** | **Test statistic** | **p-value** |  |
| Controls | 28 | 1.29 | 1.97 | 2.19 | 2.75 | 4.53 | *χ2(4) = 4.51* | 0.341 |  |
| Relatives | 10 | 1.25 | 1.44 | 2.16 | 2.64 | 4.03 |  |  |  |
| Schizophrenia | 12 | 1.53 | 2.02 | 2.66 | 3.35 | 5.02 |  |  |  |
| Bipolar | 11 | 1.54 | 1.68 | 2.03 | 2.22 | 3.38 |  |  |  |
| Schizoaffective | 11 | 1.43 | 1.63 | 2.07 | 3.31 | 7.06 |  |  |  |
|  |  |  |  |  |  |  |  |  |  |
| ***Supplemental Table 1b: Sample distribution of percept duration variance*** | | | | | | | | |  |
| **Group** | **n** | **minimum** | **lower quantile** | **median** | **upper quantile** | **maximum** | **Test statistic** | **p-value** |  |
| Controls | 28 | 0.269 | 0.710 | 1.02 | 1.69 | 5.53 | *χ2(4) = 5.34* | 0.253 |  |
| Relatives | 10 | 0.153 | 0.345 | 0.901 | 1.92 | 10.9 |  |  |  |
| Schizophrenia | 12 | 0.611 | 1.01 | 1.42 | 4.12 | 37.2 |  |  |  |
| Bipolar | 11 | 0.318 | 0.646 | 0.942 | 1.07 | 1.67 |  |  |  |
| Schizoaffective | 11 | 0.294 | 0.401 | 0.813 | 3.29 | 64.8 |  |  |  |
| **Table 1a** displays the distribution of mean percept durations within each group. **Table 1b** displays the distribution of percept duration variance within each group. In both tables, probands were divided into Schizophrenia, Bipolar, and Schizoaffective subgroups. Mean percept duration and percept duration variance were calculated for each individual as the mean and variance of their fitted lognormal distribution. Kruskal-Wallis was used to test for group differences. | | | | | | | | |  |
|  |  |  |  |  |  |  |  |  |  |


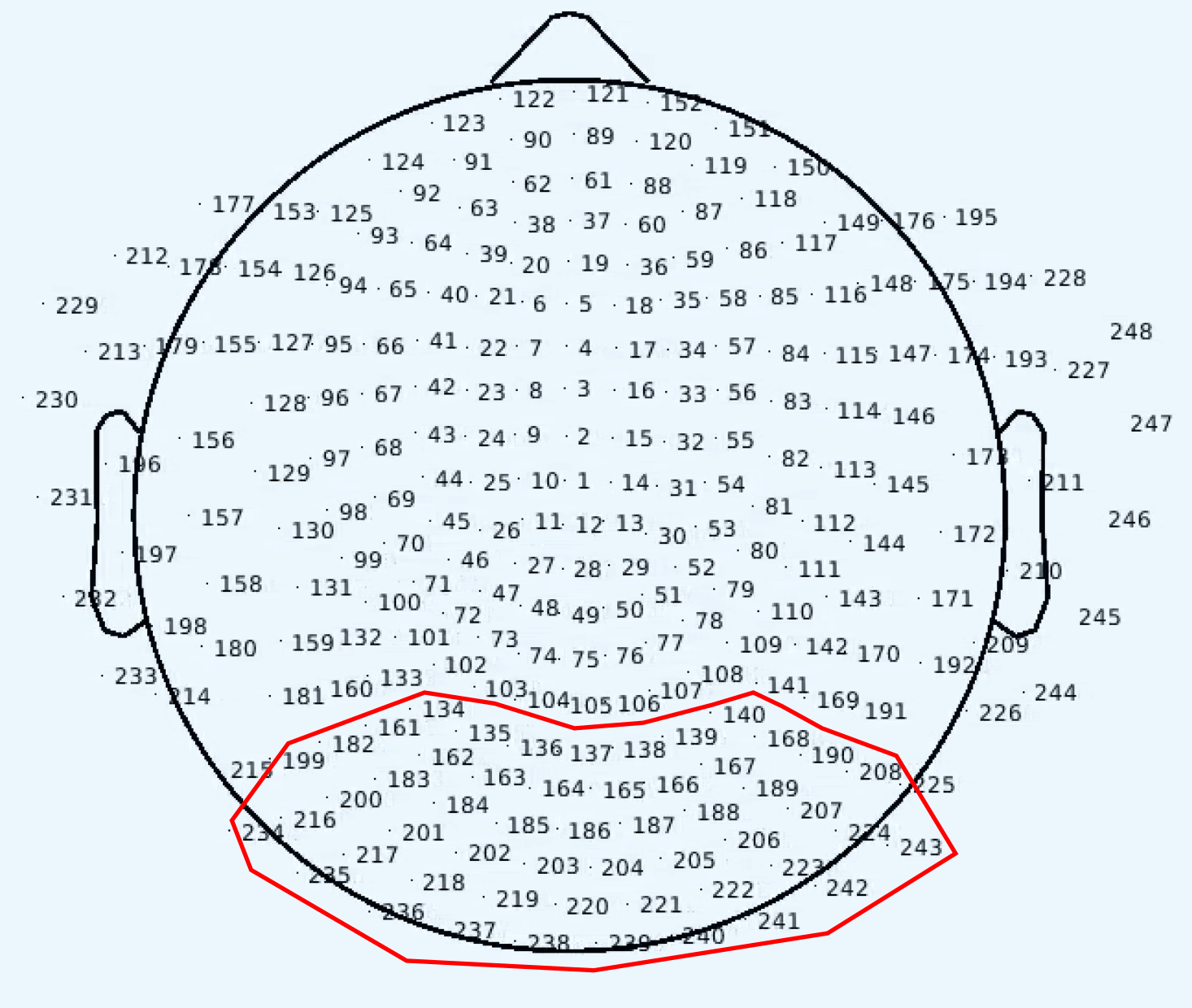


**Supplemental Figure 1. IAPF MEG Sensors.** Only sensors within the red boundary were included in the IAPF peak detection.

**
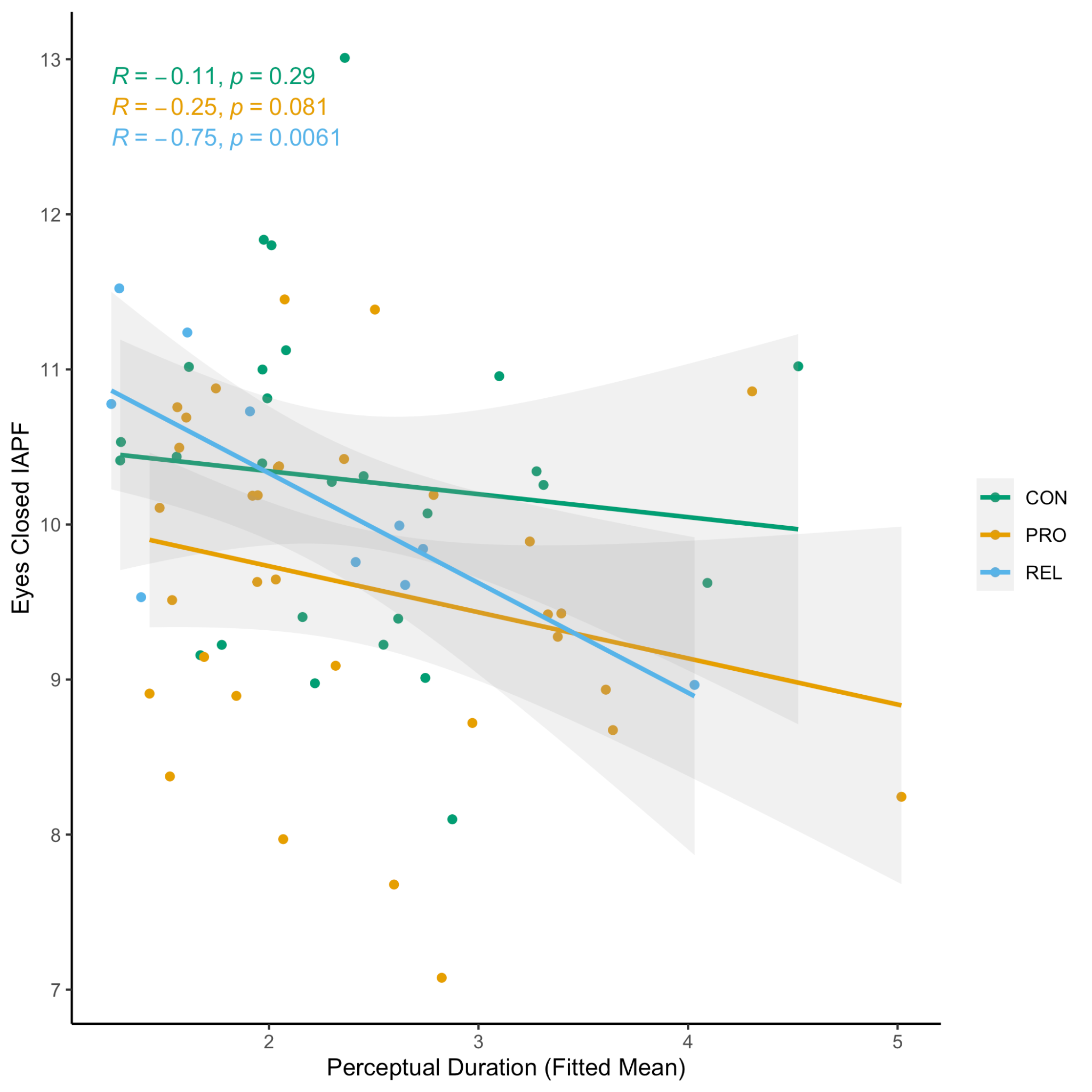

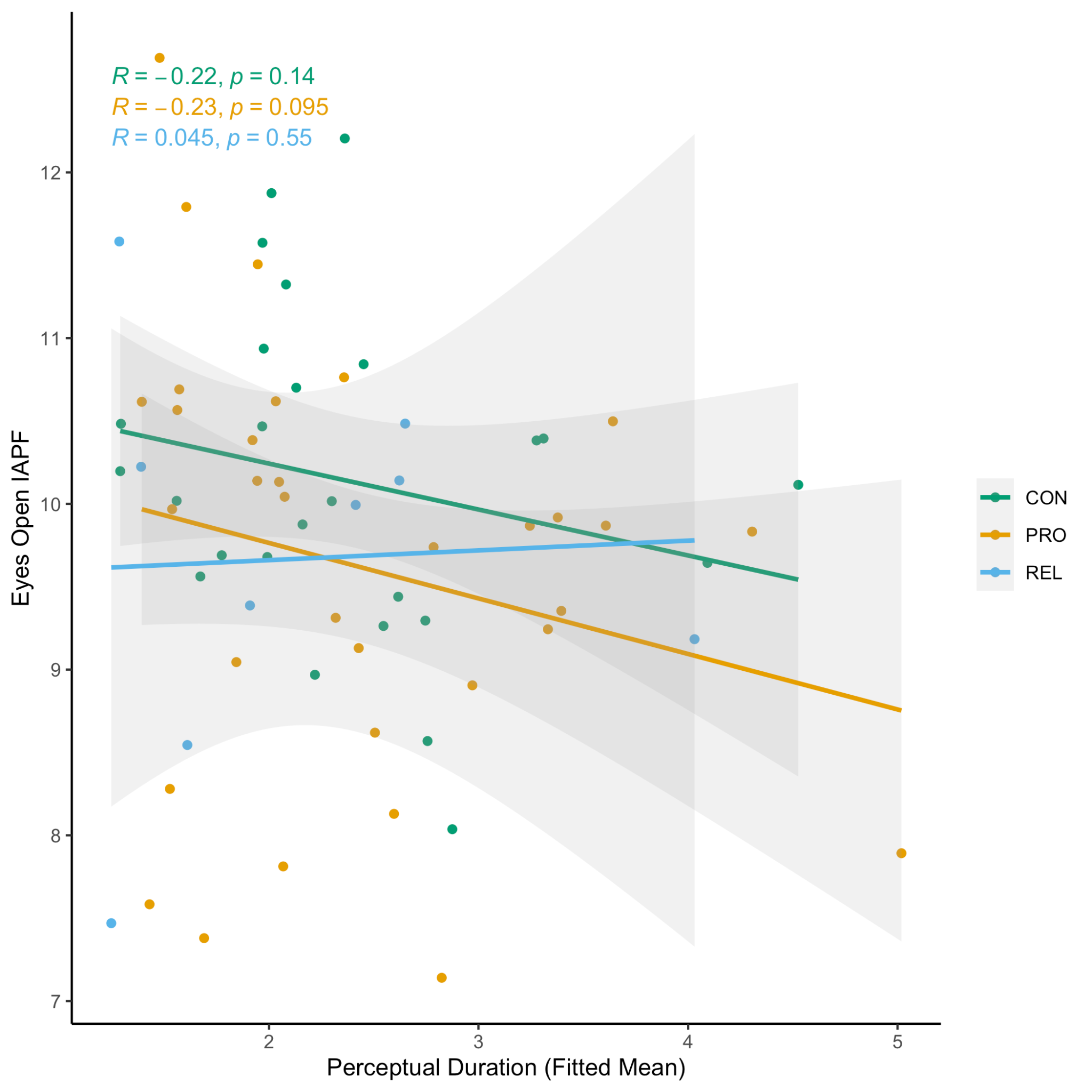
**

**Supplemental Figure 2. IAPF and Perceptual Duration.** The same data points as shown in Figure 3 are depicted, but with regression lines for each group.

**
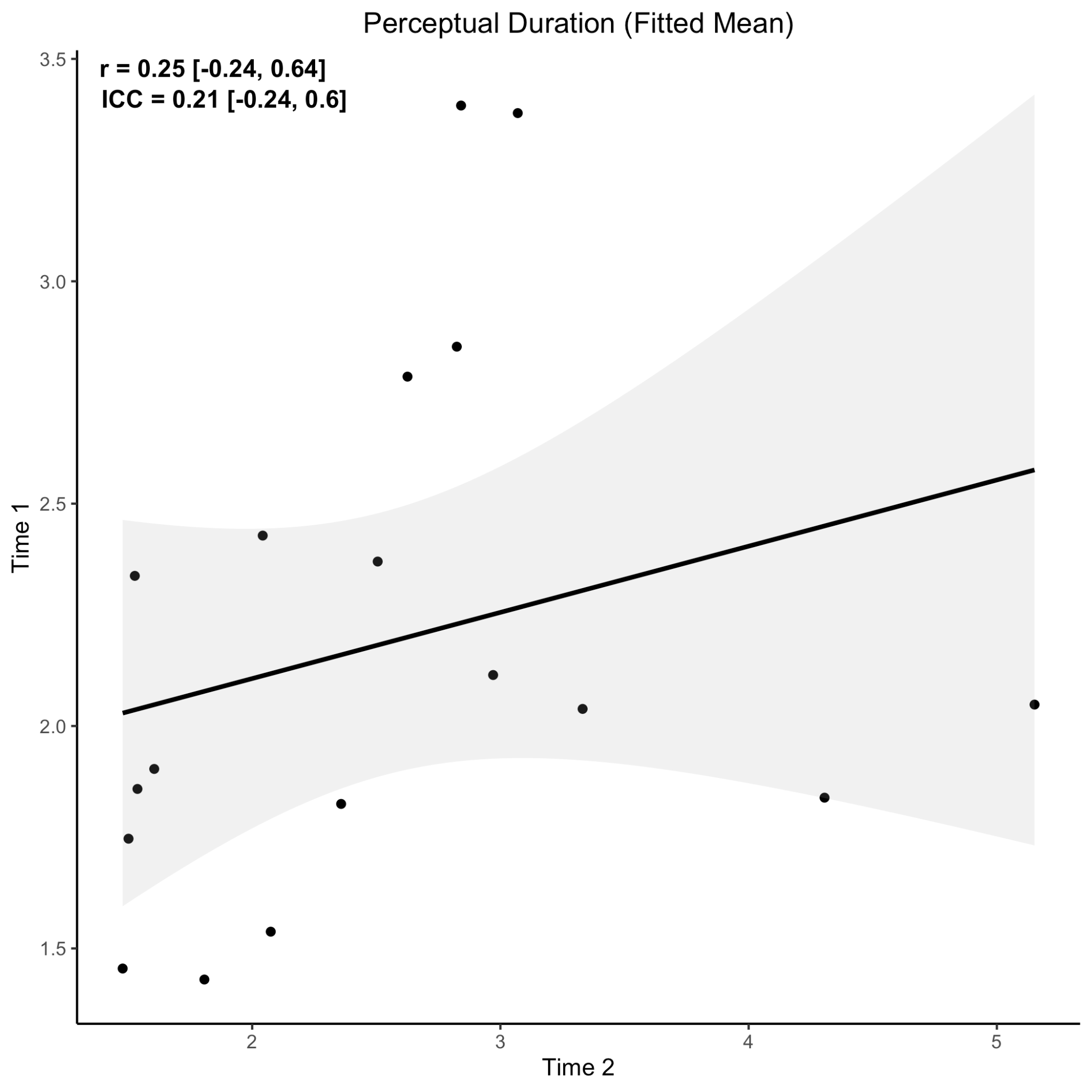
**

**Supplemental Figure 3. Perceptual Duration Test-Retest Reliability.** The same data points as shown in Figure 4 are depicted, but with a single outlier removed.


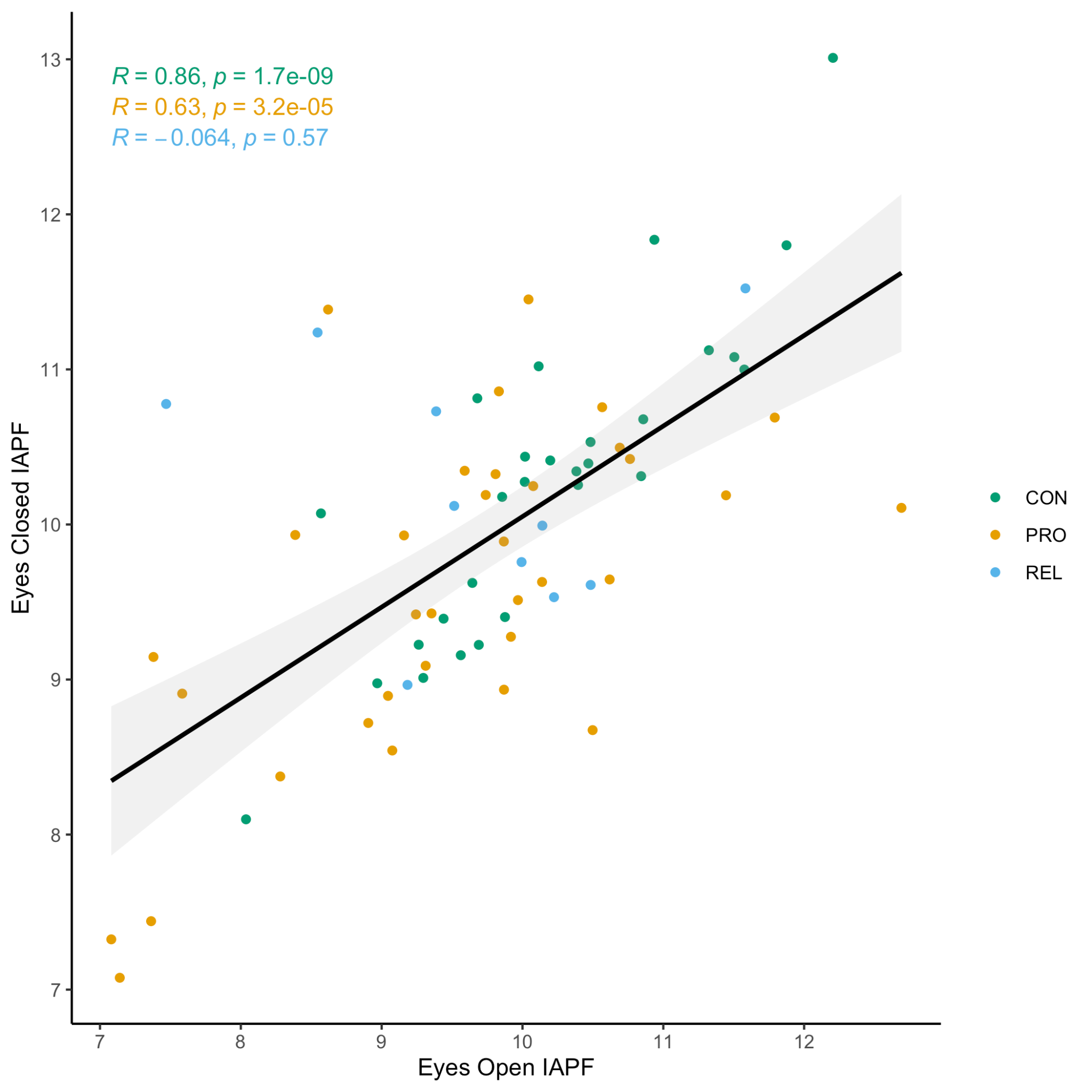


**Supplemental Figure 4. Eyes Open vs. Eyes Closed IAPF .**

**
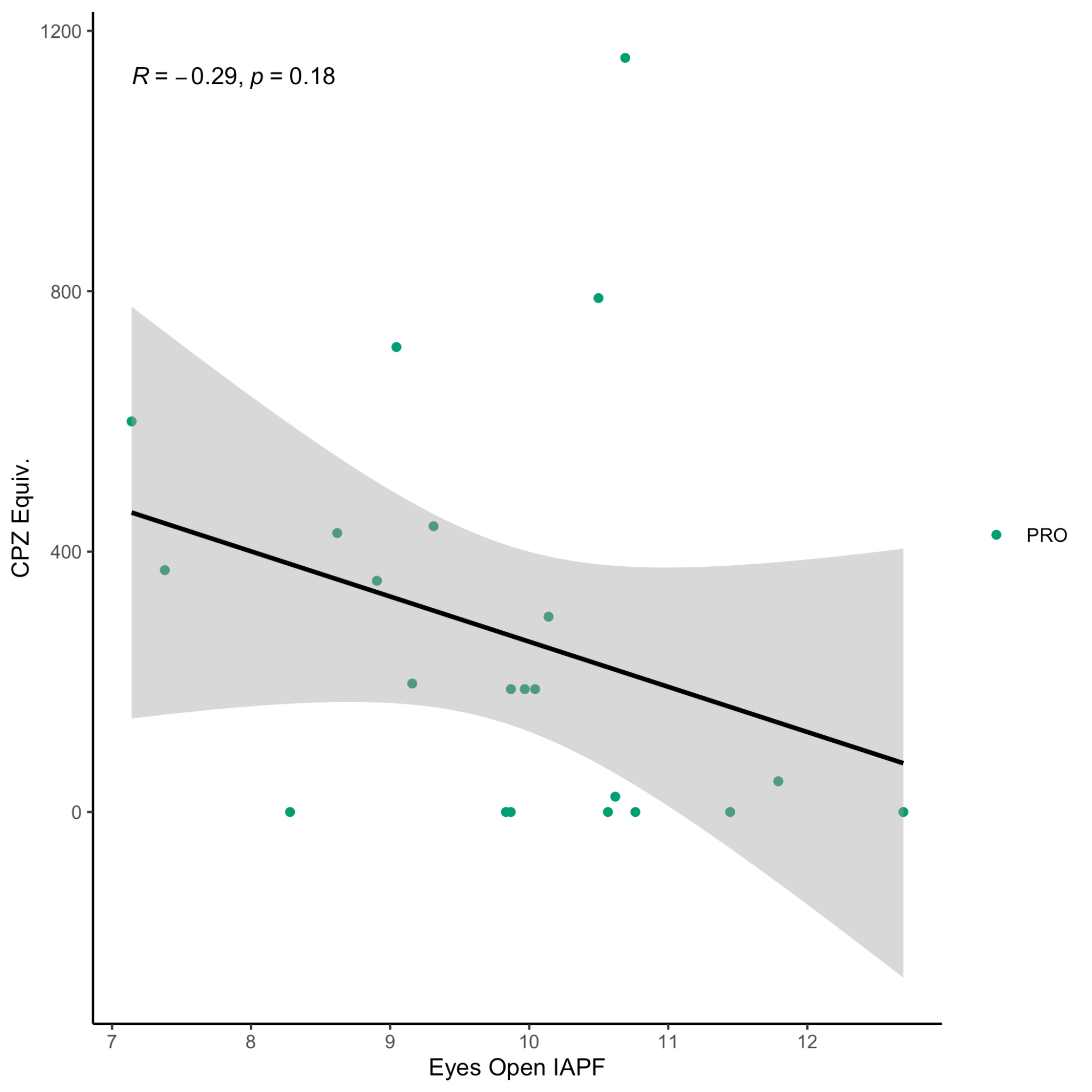

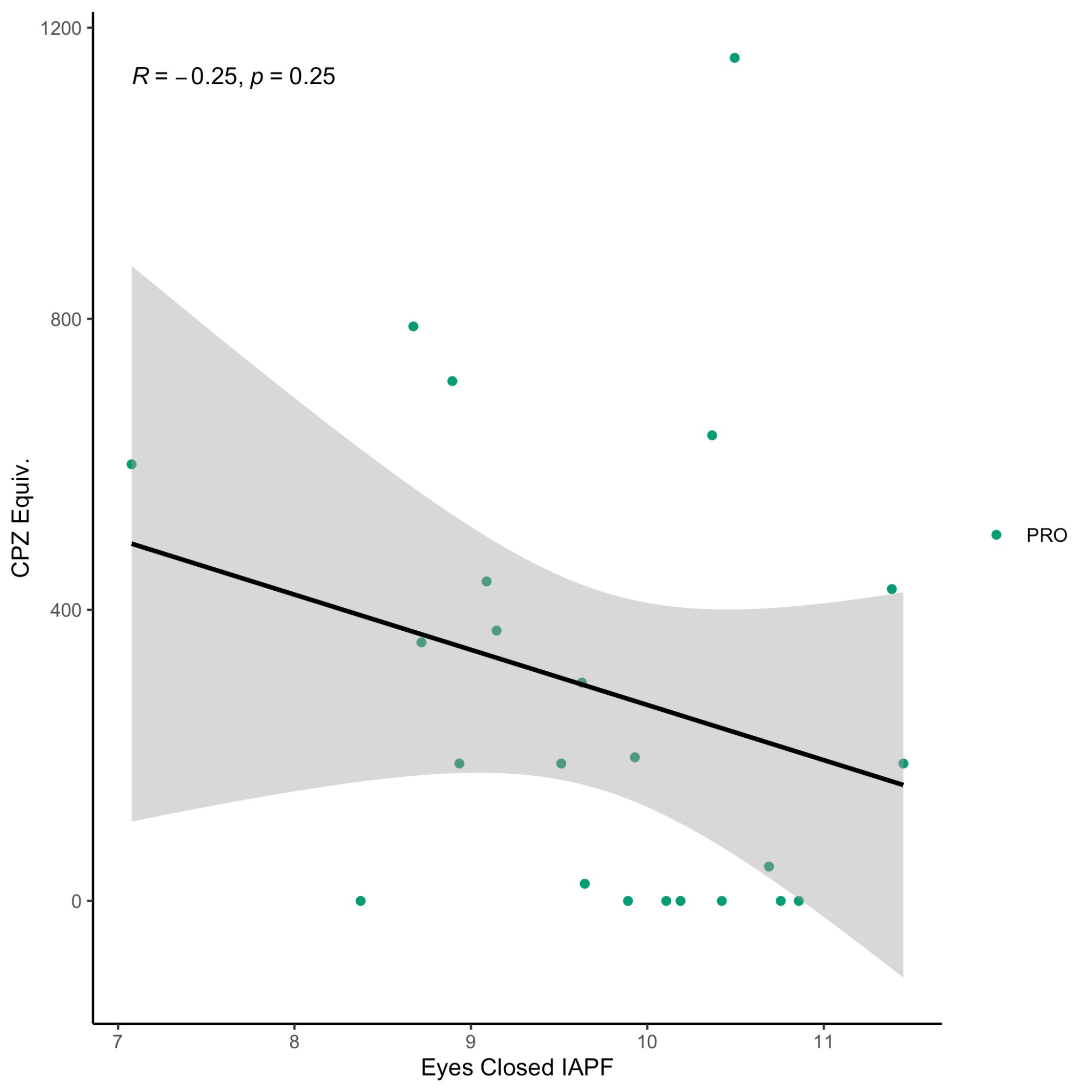

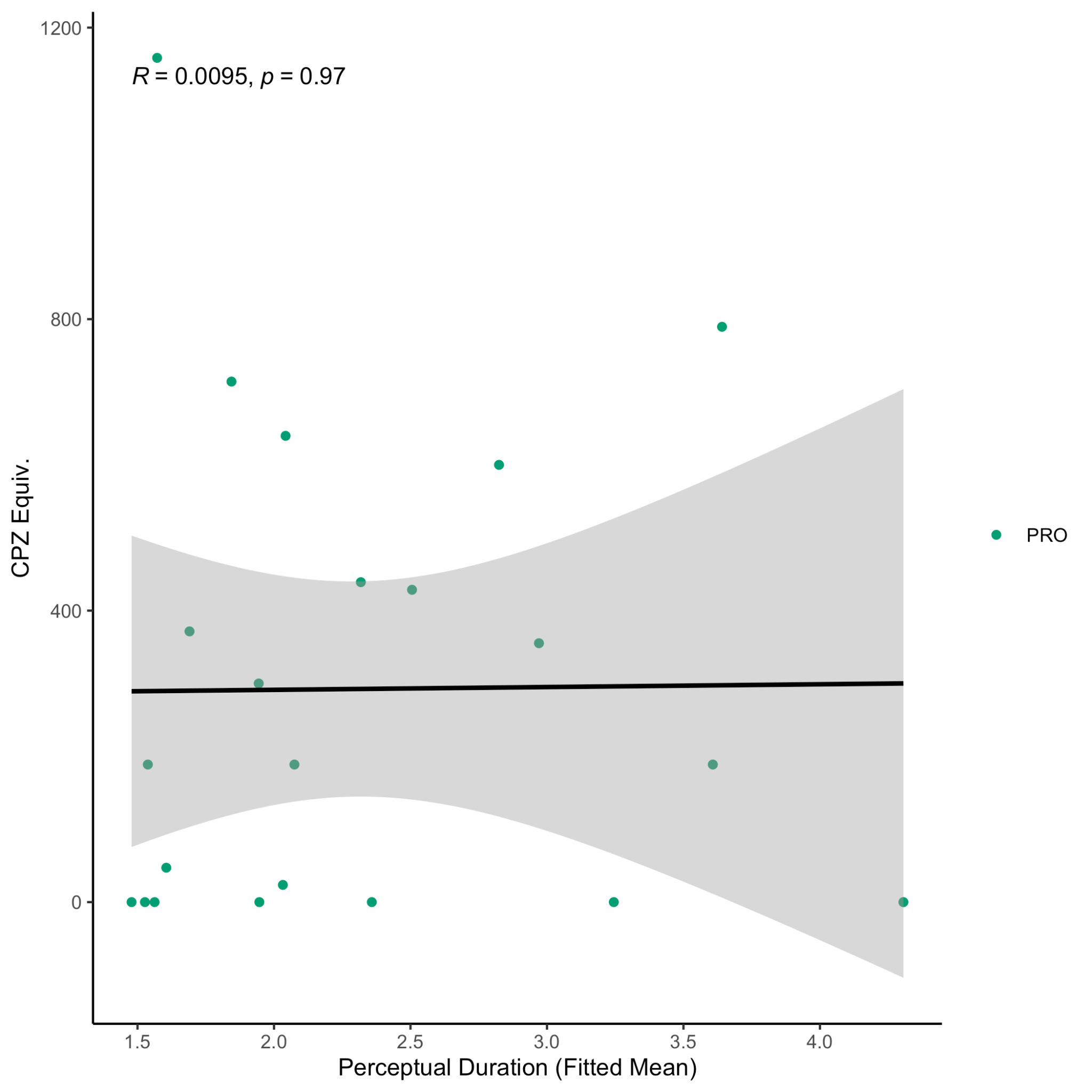
**

**Supplemental Figure 5. Relationship Between Chlorpromazine Equivalent Dosage and Primary Dependent Variables.** Y-axis is the patient’s current chlorpromazine equivalent daily dose.
